# Supplementary figures and images for: Wolbachia effects on Rift Valley fever virus infection in Culex tarsalis mosquitoes
Source: PLoS Negl Trop Dis. 2017 Oct 30;11(10):e0006050. doi: 10.1371/journal.pntd.0006050 (PMC5693443; doi:10.1371/journal.pntd.0006050)

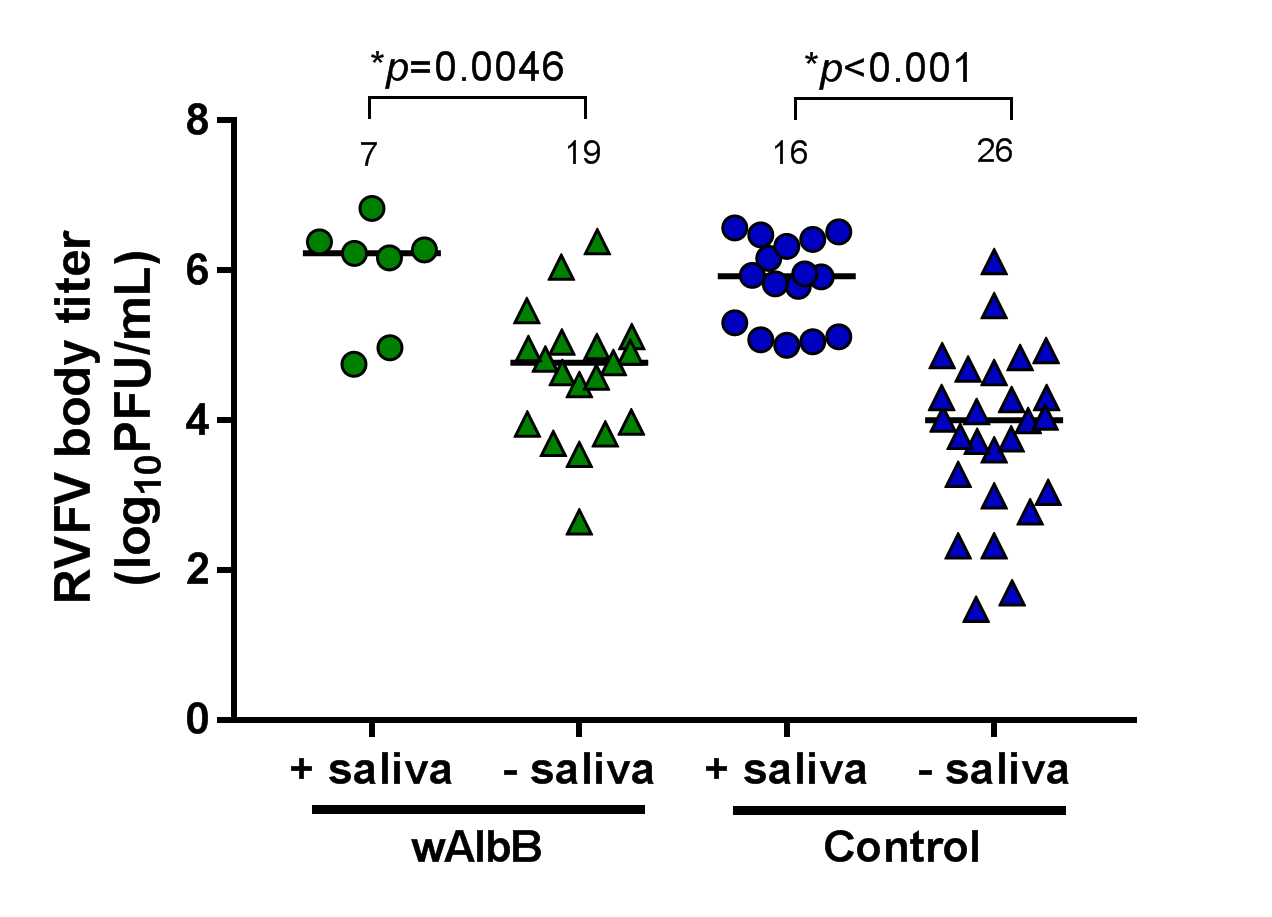

Supplement: S1 Fig — RVFV body titers were compared between mosquitoes that tested positive or negative for RVFV in their saliva. For both Wolbachia-infected and control Cx. tarsalis, mosquitoes positive for RVFV in the saliva had significantly higher RVFV body titers compared to mosquitoes negative for virus in the saliva There was no significant difference in RVFV body titer of transmitters between Wolbachia-infected and control mosquitoes (p = 0.7692). Data from three replicates were pooled and analyzed with Mann-Whitney U, and the bars represent medians. (TIF) [file pntd.0006050.s003.tif]

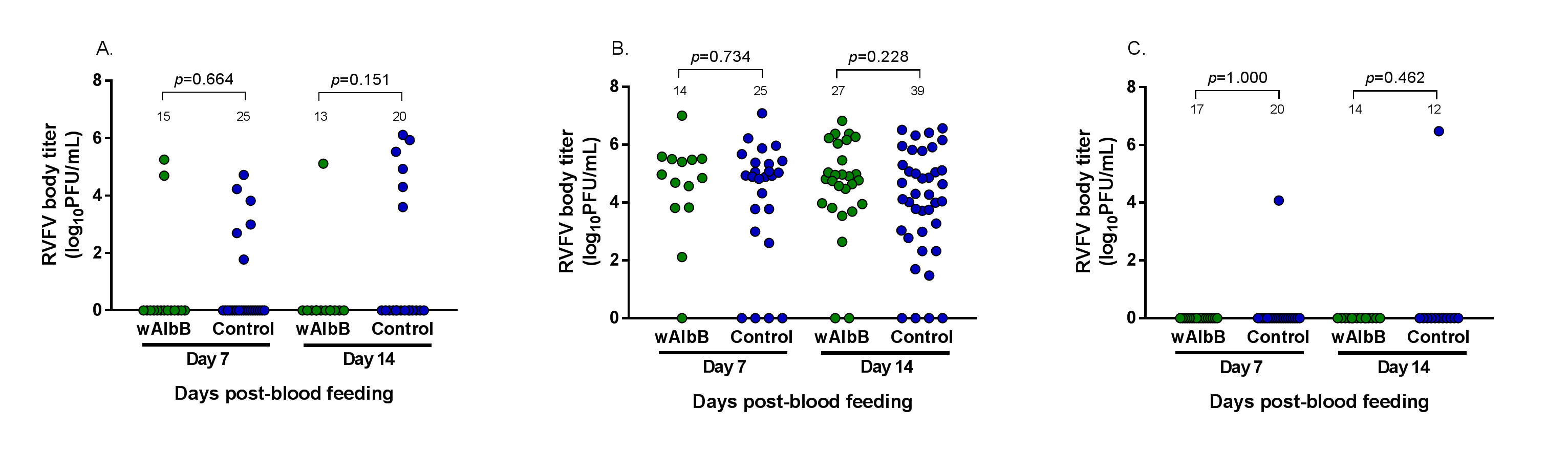

Supplement: S2 Fig — RVFV body titers were compared between Wolbachia-infected and control mosquitoes for replicates 1 (A), 2 (B), and 3 (C). In all replicates, there were no significant differences in RVFV body titer between Wolbachia-infected and control mosquitoes. Data did not pass assumptions for normality and were analyzed with Mann-Whitney U, and sample sizes are denoted above data points. (TIF) [file pntd.0006050.s004.tif]

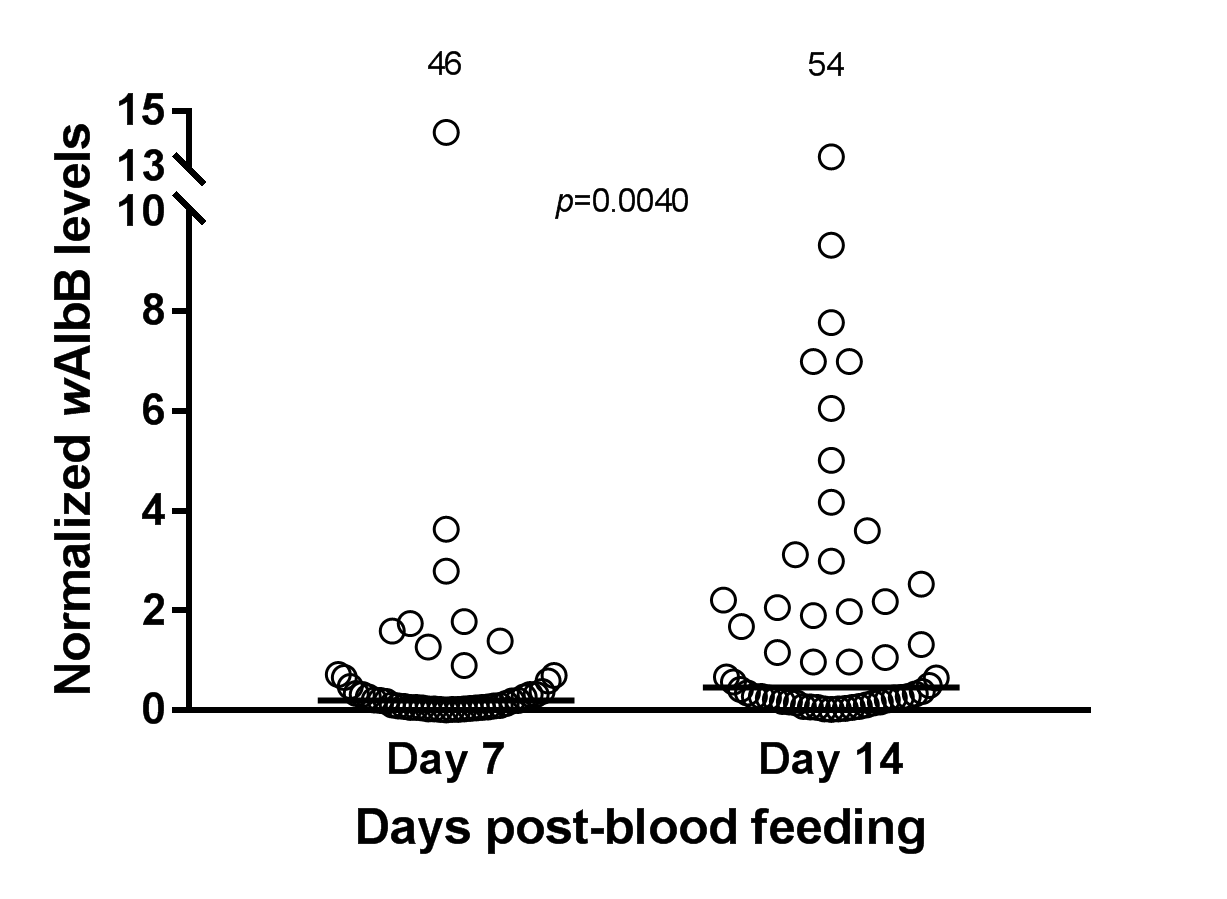

Supplement: S3 Fig — Wolbachia levels for each mosquito, determined by qPCR, were combined across all three replicates. Wolbachia levels were significantly higher at day 14 compared to day 7. Due to violations of normality, Mann-Whitney U was used for comparisons, bars are medians, and numbers above data points are sample sizes. (TIF) [file pntd.0006050.s005.tif]
